# Health for all? From lifestyle risk to genomic governance: The enduring logics of individualisation in an age of inequality

**DOI:** 10.1177/13634593261473159

**Published:** 2026-07-31

**Authors:** Paul Crawshaw

**Affiliations:** 1 Teesside University, UK

**Keywords:** genetics, lifestyle, risk and health, social inequalities in health, health policy

## Abstract

In 1997, 3 years ahead of WHO’s target of delivering Health for All, critical health scholarship was energised by the mainstreaming of social determinants and the optimism of the new public health. Yet alongside these collective ambitions, lifestyle discourses, rooted in 1970s healthism, proliferated across consumer cultures, framing health as a matter of personal responsibility. Over the past three decades, these individualising logics have intensified. Initially expressed through behavioural risk management, they now extend into molecular and algorithmic regimes, producing the ‘genomic citizen’, tasked with managing biological futures via data-driven infrastructures and market-mediated technologies. Framed as empowering, these paradigms obscure structural determinants and embed health within a political economy of anticipation, where promissory narratives justify investment while deepening responsibilisation. The COVID-19 syndemic exposed the fragility of individualised discourses, reaffirming the salience of systemic inequality. In response, this paper reflects on the concept of metabolic justice, a framework that reframes the relationship between behavioural and biological discourses while foregrounding redistribution, ecological embeddedness and systemic reform. By situating health within networks of exposure and dependency rather than individual optimisation, metabolic justice revives the ambitions of Health for All and its descendants as a materially grounded, politically transformative project for an era of social inequality and stratified medicine.

## Introduction

The centre of risk consciousness lies not in the present, but in the future (Beck, 1992: 34)

As *Health* enters its 30th year as a leading critical social science journal, this milestone invites not only celebration and reflection but also reappraisal of the radical transformations that continue to shape the terrain in which our conversations about human wellbeing, its complex determinants and governance, take place. At the beginning of the new century Robertson (2001) posited in these pages:

‘Particular discourses on health emerge at particular historical moments and gain widespread acceptance primarily because they are more or less congruent with the prevailing social, political and economic order within which they are produced, maintained and reproduced’ (pp. 294–295).

More than two decades ago, Robertson’s (2001) analysis was pivotal in shaping my own doctoral work on men’s health (Crawshaw, 2007). Her observation remains apposite. Health and medicine, as Virchow (1958) insisted, are fundamentally social concerns, bound up with questions of justice, governance and the organisation of human life and determined by the political and economic conditions that shape the everyday experiences and expectations of populations. At the turn of the century, the World Health Organisation’s (WHO) programme *Health for All* was central to efforts to address the social and political determinants of health, not as a neutral aspiration, but as a radical, redistributive vision rooted in the politics of justice, equity and structural reform (Kickbusch, 2003). The emergence of *Health* as a critical interdisciplinary journal can be understood as part of this same political and intellectual movement, one in which questions of governance, inequality and the social determinants of health have been foregrounded across both policy and scholarship.

The year 2000 marked the nominal end of *Health for All’s* original goal, but its principles have continued in frameworks such as Universal Health Coverage (UHC) and the Sustainable Development Goals (SDGs), which foreground equity, access to health services and social justice (WHO, 2025). Despite continuing aspirations for structural and systemic change, the intervening decades have seen powerful political and cultural forces associated with neoliberalism reframe health not as a collective project but as a personal one. This tension is particularly striking as we mark 40 years of the *Health for All* agenda (Shiroya et al., 2026). With roots in the 1970s (Crawford, 1980), such neoliberal rubrics position good health as a universally attainable goal, with individuals increasingly tasked with risk-literate management of behaviour, opportunity and self-optimisation within market logics increasingly mediated by emerging biomedical technologies (Rose, 2007).

Adopting a critical genealogical approach, this paper draws on key texts in medical sociology and health studies to trace the shifting logics through which health has been conceptualised and governed. It synthesises existing literature to examine how behavioural, molecular and emerging paradigms reproduce and reconfigure relations of responsibility, governance and inequality. Analysis focuses primarily on developments in high-income, Global North contexts, while recognising that these dynamics are uneven and context specific. A key aim is to examine how contemporary health discourses reflect and reproduce prevailing political, economic and social logics (Robertson, 2001) and how these in turn shape both the micro politics of health behaviours and wider systemic questions of health equity (Fox, 2024). The implications for reclaiming the values underpinning collective visions such as *Health for All* and its descendants, along with their radical and redistributive ambitions, are a central focus.

## The political economy of health governance

The late 20th century was characterised by a hegemonic narrative of consensus and optimism, grounded in the ascendancy of neoliberal political and economic ideology. This narrative positioned liberal democracy and market capitalism as both triumphant and globally expanding, underpinned by cooperative forms of governance and a rules-based international order (Harvey, 2005; Mounk, 2020). In this imagined consensus, *Health for All* gained traction, congruent with assumptions that rising prosperity and cooperative globalisation would enable poorer nations to converge with the wealthy through targeted interventions and structural reform. This was to take place against an economic backdrop of unfettered market capitalism (Harvey, 2005) and accelerated integration, presented as capable of delivering universal progress and narrowing global disparities. Events in the decades that followed rapidly unsettled these assumptions.

Early in the new century, the fragility of this imagined global consensus was exposed by geopolitical shocks, including the 9/11 attacks, subsequent invasions of Afghanistan and Iraq and the rise of new antagonisms, including transnational political violence, post-Soviet ethno-political conflicts and intensifying US–China rivalry (Record, 2008). These and other tensions fractured illusions of stability whilst revealing the limits of liberal internationalism (Gontijo, 2025). Despite claims that we had arrived at the end of history (Fukuyama, 1991), the Cold War’s East–West binary persisted, refracted less through economic ideology than through cultural, religious, and civilisational antagonisms that reshaped global fault lines. At the same time neoliberal capitalism consolidated its dominance, embedding the mantra of there is no alternative (Harvey, 2005), systematically marginalising dissenting models, particularly those rooted in non-western epistemologies and political traditions. The result was a rapid decollectivisation of welfare, following a shift towards advanced liberalism as a model for governing late modern democracies, privileging individual responsibility, market rationality and technocratic control.

Within this shifting terrain, shaped by consolidation of neoliberalism as *the* dominant socio-political rationality, health emerged as a key site for the enactment of these logics (Robertson, 2001). Under conditions of what Fisher (2009) terms ‘capitalist realism’, collective approaches and solidaristic organisation were steadily displaced by lifestyle discourses that framed individuals as agents of risk and productivity (Laruffa, 2022). These discourses reinforced the global dominance of Western biomedicine, while promoting digital health technologies and intensified individualisation of care and governance (Baru and Mohan, 2018). Despite earlier optimism about global convergence, such developments mirrored and extended broader neoliberal modes of social organisation (Robertson, 2001), raising urgent questions about the viability of progressive health projects in an era marked by deepening inequality and growing conflict. Global governance initiatives such as the SDGs have continued to perform commitments to equity and social justice, yet these aims are frequently undermined by the continuing dominance of capitalist logics, which privilege competition over collectivism and market logics over solidarity (Harvey, 2005). This contradiction is captured by Hasan (2025), who, following Fisher (2009), characterises it as the ‘tragedy’ of capitalist realism.

Contemporary health discourses unfold against this backdrop of contradiction, shaped by persistent tensions between individual responsibility and the structural and systemic determinants of wellbeing. Here, neoliberal logics actively construct an autonomous, self-managing subject, tasked with navigating risk and optimising productivity under the guise of informed choice. In doing so, these logics obscure the enduring influence of social, economic, and political structures on health outcomes, reframing inequality as a matter of personal failure rather than systemic constraint. Expertise and lay knowledge intersect and often collide as individuals navigate increasingly complex health landscapes mediated by digital technologies, algorithmic diagnostics (Ajana, 2020), genetic histories and personalised medicine. While these developments are framed by governments and industry alike as empowering, they raise critical questions about agency, equity and resource distribution. These tensions demand renewed attention to how health is governed, experienced, and contested in everyday life, where lifestyle, risk and governance intersect in broader struggles over the meaning and ownership of health (Bell, 2015).

This paper critically examines how responsibility for health has been framed over the past three decades, tracing the convergence of lifestyle discourses from the 1970s with new discovery science in genomics, bio-medical innovation and neoliberal forms of governance. Despite global efforts to achieve equity, health has been further individualised through the rise of lifestyle medicine, genomics and personalised biomedical technologies that position the body as a site of optimisation, moral regulation and market engagement (Brown, 2013; Olurunsogo et al., 2024). Drawing on sociological critiques of risk, responsibilisation and governance, this paper interrogates the cultural and political logics that have sustained this individualisation, showing how dominant paradigms displace structural determinants and reframe inequality as personal failure. These processes are situated within a political economy of health innovation, where promissory narratives (Tutton, 2014) of technological progress work to justify investment and growth while eliding questions of redistribution and access. At the same time, the depoliticisation of public health policy, through behavioural interventions (Crawshaw, 2013) and technocratic governance continues to obscure the social conditions of illness, entrenching models of health that privilege individual agency over collective responsibility. This analysis reasserts the relevance of critical health scholarship in an era marked by widening inequalities (Bambra, 2024), the syndemic revelations of COVID-19 and the intensifying contradictions between collective vulnerability and individualised health governance (Crawshaw and Gray, 2026). Revisiting key theoretical debates and empirical developments, it asks what it means to pursue collective ambitions in a context where health is increasingly technologised, commodified and governed through logics that obscure its structural determinants.

## Lifestyle, risk and the responsible subject

In the post-war Western democracies, health was established as a collective responsibility, delivered through state-led infrastructures with governments positioned as guarantors of population wellbeing (Manow, 2021). In the UK, this logic was institutionalised through the creation of the National Health Service (NHS) in 1948, while the founding of the WHO (1946) signalled health as a global concern grounded in the right to the highest attainable standard of health. Initiatives such as Alma Ata (1978) extended this vision, foregrounding equity, primary care and structural reform (Davletov et al., 2018).

Despite variations across contexts, this model broadly positioned health as a social good shaped by collective provision and structural conditions. However, by the final third of the 20th century, this settlement had begun to fragment. Alongside the partial privatisation of public systems (Pollock, 2000), health became increasingly dedifferentiated (O’Brien, 1995), extending beyond clinical institutions into everyday life. Influenced by risk factor epidemiology, behavioural science and consumer culture, the self emerged as a site of health management (Crawshaw, 2007). Health was no longer an episodic concern but a continuous project, reflexively monitored and optimised through everyday practices (Petersen and Lupton, 1995).

These shifts were closely linked to changes in disease patterns. As infectious diseases declined in high-income contexts, chronic conditions such as heart disease and diabetes rose, reorienting public health priorities towards prevention and risk management (Krieger, 2011). Epidemiology responded by translating diffuse causation into calculable risk, reframing behaviours such as smoking, diet and exercise as modifiable factors subject to intervention (Krieger, 2011). In this context, probabilistic reasoning became central to public health, embedding prevention within strategies of behavioural governance.

Rose’s concept of the prevention paradox crystallised this logic, demonstrating how small shifts in risk across populations could produce substantial collective gains (Rose, 1993). Such reasoning underpinned interventions including smoking bans, dietary guidelines and screening programmes, embedding prevention within everyday life. While framed as evidence-based and cost-effective, these approaches also reinforced responsibilisation, positioning individuals as agents of risk management within broader regimes of governance.

Lifestyle risk thus emerged not merely as a technical response to chronic disease, but as a normative project that recast health as a matter of personal responsibility. By translating complex causation into calculable risk, it extended medical authority into everyday life and laid the foundations for a new healthism (Crawford, 1980) alongside surveillance medicine (Armstrong, 1995). The result was the emergence of a responsibilised subject, tasked with managing uncertainty and optimising health through continuous self-regulation (Crawshaw, 2007).

What began as a technical project of risk factor epidemiology evolved into broader regimes of surveillance. As Armstrong (1995) shows, surveillance medicine expanded the scope of medical authority to encompass the well, establishing health as an anticipatory regime of continuous monitoring and intervention. These developments blurred the boundaries between sickness and wellness, embedding risk within everyday life and aligning governance with neoliberal rationalities that privilege personal responsibility over structural provision.

Healthism provided the normative force for this shift, framing health as a moral and civic obligation (Crawford, 1980). Individuals were encouraged not only to adopt healthy behaviours but to internalise expert knowledge and engage in continuous self-surveillance, translating health into a project of optimisation and worth. As Armstrong’s (2025) analysis demonstrates, the concept has proven highly adaptable, extending across contexts from lifestyle medicine to genomics and algorithmic diagnostics. Rather than being displaced, healthism has been reconfigured, embedding responsibilisation within new biomedical paradigms.

If healthism supplied the normative force for individualised health governance, epidemiology and behavioural science provided its operational machinery. Together, they constructed the healthy subject as disciplined, self-monitoring and accountable, aligning everyday conduct with broader regimes of governance (Bell, 2015). Crucially, these developments did not mark a rupture with earlier paradigms but established the conditions for their extension. Lifestyle governance provided the foundation upon which molecular forms of optimisation would later build.

The epidemiological transition thus laid the groundwork for contemporary health governance, embedding prevention, risk and self-management at its core. These dynamics aligned closely with neoliberal rationalities, reframing wellbeing as a moralised project of individual responsibility while displacing structural determinants. In doing so, they prepared the ground for their molecular recalibration in genomic medicine, where responsibility shifts from behavioural optimisation to the anticipatory governance of biological futures.

## From lifestyle to life itself: Molecular governance and the genomic citizen

Over the past three decades, lifestyle discourses have not only persisted but intensified. They have also extended into domains such as aesthetic health, where body modification and cosmetic procedures are normalised as forms of optimisation in the pursuit of beauty and wellbeing (Featherstone, 1982). Contemporary developments in wellness culture (Baker, 2022) and aesthetic medicine illustrate this shift, with interventions such as minimally invasive procedures and body-shaping technologies positioned as extensions of health rather than cosmetic enhancement (Andrus, 2024; Rhodes, 2024).^1^ Such developments reflect a broader fusion of health and consumer culture, in which practices of optimisation, self-monitoring and discipline become markers of value (Ajana, 2020). Digital technologies further embed these logics within everyday life. The expansion of self-tracking, datafication and AI-enabled systems intensifies the inscription of health within digital infrastructures (Lupton, 2026). These platforms and wellness applications encourage continuous monitoring and the performance of ‘affective labour’, reinforcing individual accountability within neoliberal market logics while simultaneously generating new forms of inequality and harm (Kent, 2023). Concurrently, new biomedical paradigms extend these individualising imperatives into the molecular domain, where health is governed through genomic profiling, algorithmic diagnostics and predictive analysis (Kent, 2023; Rose, 2007). This molecular turn marks a shift from managing behaviour to monitoring biology, as genomic and data-driven interventions promise increasingly precise forms of prediction and control (Rasooly et al., 2022). Rose (2007) characterises this transformation as the emergence of a ‘politics of life itself’.

If lifestyle discourses framed health as a behavioural project of self-management, molecular biomedicine introduces a new layer of governance operating through biology rather than behaviour. Central to Rose’s (2007) account is the notion of somatic individuality, through which responsibility is inscribed in the molecular constitution of bodies and individuals are tasked with managing biological futures via genomic profiling and algorithmic diagnostics (Dominiczak et al., 2025; Tutton, 2014). Although centred on the body rather than behaviours, this recalibration does not dismantle the responsibilisation characteristic of lifestyle discourses. Importantly it does not constitute an epistemological rupture (Bachelard, 1984). Rather, it presents continuation and intensification, embedding self-optimisation within the fabric of life itself. Where the subject of lifestyle governance is encouraged to manage risky behaviours, the genomic citizen is tasked with interpreting probabilistic data, managing predispositions and engaging with biomedical interventions. Together, these developments extend earlier logics of individualisation, prefigured in the work of Petersen and Bunton (2002), and remain congruent with neoliberal forms of health governance that prioritise responsibility, optimisation and self-management (Robertson, 2001). The risk is that what appears as empowerment is, in practice, an intensification of neoliberal rationalities.

What Tutton (2014) terms promissory medicine sits at the heart of this shift. Complementing Rose’s (2007) account of somatic individuality, Tutton shows how anticipatory regimes mobilise imagined futures of precision, longevity and empowerment as justificatory devices for present investment, participation and compliance. Together, these frameworks reveal how responsibility is simultaneously inscribed at the molecular level and projected forward through anticipatory logics, embedding neoliberal imperatives of optimisation within both biological futures and everyday decision-making. In this sense, promissory medicine does not mark a departure from lifestyle governance but represents its reconfiguration, translating behavioural responsibilisation into the anticipatory management of biological risk. These narratives underpin a political economy of anticipation in which visions of personalised care obscure structural determinants and inequalities in access, relocating responsibility from social systems to individual bodies. In doing so, they intensify the moralisation of health and embedding it as a continuous object of calculation and optimisation.

Taken together, these developments extend earlier logics of lifestyle governance into a molecular register. The genomic citizen is not a departure from the responsibilised subject, but its reconfiguration, governed through data, anticipatory frameworks and market logics that embed responsibility within biological futures.

This continuity is further illuminated by Armstrong’s (2019) genealogy of diagnosis, building on his earlier analysis of ‘surveillance medicine’ (Armstrong, 1995). In this account, diagnosis shifts from pathology-based classification to predictive regimes in which prognosis increasingly shapes intervention. Molecular governance extends these earlier forms of surveillance medicine, embedding anticipatory logics within everyday health practices and relocating intervention from the management of present illness to the governance of probabilistic futures. These developments are most clearly operationalised in contemporary regimes of precision medicine and data-driven health governance. In this trajectory, from surveillance medicine’s expansion of medical authority into everyday life to predictive diagnostics and promissory regimes, individuals are enrolled not only as patients but as active participants in data-driven systems of governance (Armstrong, 2019; Tutton, 2014). Health is increasingly reframed as a continuous object of calculation, stratification and optimisation, a logic that finds its most intensive expression in personalised and precision medicine. As Armstrong’s account of predictive diagnostics suggests, this progression is not a series of ruptures but a deepening of anticipatory governance in which prognosis rather than pathology drives intervention. These dynamics culminate in algorithmic infrastructures and precision medicine, where stratification and prediction are operationalised in practice, as explored in the following section.

## Molecular governance and the politics of promissory medicine

Building on this trajectory, developments in personalised and precision medicine exemplify how anticipatory governance is operationalised in practice, embedding responsibilisation within molecular and algorithmic infrastructures (Dominiczak et al., 2025). These paradigms promise to tailor care to genetic and molecular profiles, positioning health as a continuous object of calculation and optimisation. Situated at the intersection of academia, industry and healthcare, they integrate genomic, digital and algorithmic technologies into clinical practice, not only predicting risk but actively stratifying populations through data-driven segmentation and personalised intervention (Lupton, 2026).

This logic of stratification is increasingly embedded within clinical systems. In oncology, for example, treatment pathways are now organised around genomic profiling. In non–small-cell lung cancer (NSCLC), patients with activating EGFR mutations are prescribed targeted therapies such as third-generation tyrosine kinase inhibitors, while others remain on standard chemotherapy regimens (Zhou et al., 2025). In such cases, genomic information can materially shape therapeutic outcomes for particular subpopulations. However, such benefits remain unevenly distributed and contingent on access to advanced diagnostic infrastructures, while doing little to address the structural determinants of disease. At the same time, promissory narratives surrounding precision medicine frequently exceed its current clinical capacities, framing speculative futures of precision and control as present realities.

These developments are further intensified through algorithmic infrastructures that aggregate genomic, proteomic and behavioural data, enabling predictive modelling and large-scale population segmentation (Green et al., 2023). These systems do more than support clinical decision-making; they constitute sites of governance within digital architectures, enrolling individuals as both data producers and consumers within anticipatory regimes of health management. As Peng (2026) argues, the material outcome is that they do not simply identify pre-existing medical realities but actually construct normative frameworks of health, embedding assumptions about risk, value and worth that reflect and reproduce existing social inequalities. Through these infrastructures, stratification becomes a continuous process, as populations are segmented, monitored and managed according to probabilistic risk profiles. Recent public health commentary similarly suggests that such developments risk diverting attention from population-based strategies embedded in the social determinants of health, with reliance on individualised interventions framed as a potential ‘societal failure’ (Webster and Ghebrehewet, 2026).

National policy initiatives illustrate the institutionalisation of these dynamics. The NHS’s Accelerating Genomic Medicine strategy, for instance, seeks to embed whole-genome sequencing within routine care alongside integrated data platforms and machine-learning tools (NHS England, 2022). These developments exemplify the ‘triple helix’ of academia, industry and healthcare, positioning precision medicine not only as a paradigm of care but as a driver of economic innovation (Dominiczak et al. (2025). Here, the optimisation of life converges with the capitalisation of health. In this context, genomic knowledge operates both as a clinical resource and as an economic asset, reinforcing the market-oriented logics that underpin contemporary health governance.

Although often presented as holistic and patient-centred, personalised medicine frequently reconfigures earlier ideals of individualisation through molecular and data-driven logics. As Tutton (2014) suggests, it operates within a political economy of anticipation in which promises of precision and empowerment justify investment and participation while obscuring structural determinants. Under this model, responsibility is increasingly relocated from social systems to individual bodies, intensifying pressures towards optimisation while leaving existing inequalities largely intact. Risk is no longer framed as behavioural alone but inscribed within the genome and rendered actionable through predictive modelling, encouraging individuals to monitor predispositions and pre-empt disease through biomedical consumption. Framed in these terms, molecular governance deepens rather than displaces earlier regimes of lifestyle risk, translating behavioural responsibilisation into forms of genomic optimisation and intensifying the moral imperative of health. However, the limits of these individualising and anticipatory frameworks raise critical questions about how health might be re-centred as a collective, and structurally grounded project.

## The return of inequality: COVID-19 and its syndemic revelations

While recent decades have been dominated by paradigms that individualise responsibility through lifestyle modification and, more recently, genomic optimisation, the enduring influence of structural determinants continues to expose the epistemological limits of these approaches. Such evidence not only challenges the normative dominance of responsibilisation but foregrounds the political economy of health as a decisive force in shaping vulnerability, disrupting promissory narratives of empowerment and demanding systemic reform. Evidence is compelling that poverty, housing and labour conditions exert a profound and cumulative influence on morbidity and mortality. These structural forces are not only obscured by individualised paradigms, but actively depoliticised, reframing systemic vulnerability as a matter of personal failure (Bambra et al., 2021; Marmot and Allen, 2025). At the same time, widening socioeconomic inequality has re-emerged as a central concern in academic and policy debates. Savage (2021) documents the resurgence of class stratification in the UK and beyond, while Piketty (2014) traces a U-shaped trajectory of wealth disparities since the 1980s, a structural pattern that provides the backdrop for widening health inequalities. These shifts are reflected in persistent mortality gaps and declining life expectancy among disadvantaged communities, underscoring the structural violence embedded in health outcomes (Marmot and Allen, 2025).

The COVID-19 pandemic rendered these dynamics starkly visible. Contrary to narratives portraying the virus as an equalising force (Mein, 2020), the pandemic operated as a syndemic (Horton, 2020), in which infection interacted with and amplified pre-existing inequalities in income, housing, employment, racialised disadvantage and comorbidity (Bambra et al., 2021). This pattern is evident across multiple contexts. In Minnesota, for example, COVID-19 mortality reached 459 per 100,000 in the most deprived neighbourhoods, over 3.6 times higher than the 126 per 100,000 observed in the least deprived areas (Wrigley-Field et al., 2021). Across US counties, the most deprived decile registered 233 excess deaths per 100,000 compared to the least deprived counties (Dukhovnov and Barbieri, 2022). In England, mortality rates were approximately double in the most deprived areas overall, and among those under 65 nearly 3.7 times higher between March 2020 and March 2021 (Barnard et al., 2021; Suleman et al., 2021).

These dynamics did not emerge in isolation but were layered onto decades of widening socioeconomic inequality. Wealth and health disparities intensified following the neoliberal turn of the late 1970s (Harvey, 2005), accelerating through the 2010s (Savage, 2021) as governments responded to the 2008 financial crisis with austerity measures. These measures dismantled welfare infrastructures, deepened labour precarity and eroded collective forms of social protection.

Long COVID further illustrates how these syndemic processes extend beyond acute infection, compounding pre-existing disadvantage through persistent illness. In the United States, 7.2% of adults previously infected with COVID-19 reported Long COVID by 2022, disproportionately affecting those experiencing financial hardship, food insecurity and limited access to healthcare. Globally, longitudinal evidence suggests enduring impacts among children, including fatigue and reduced physical activity (Ding et al., 2025). These patterns highlight how chronic conditions interact with socioeconomic stressors, deepening inequality across life courses and generations.

Collectively, the syndemic revelations of COVID-19, alongside persistent poverty, housing precarity, labour exploitation and ecological harms, expose the limits of individualised paradigms and reassert the centrality of structural determinants in shaping health (Bambra et al., 2021; Horton, 2020; Marmot and Allen, 2025). Population health outcomes emerge not as the product of individual behaviour or genomic predisposition alone, but the cumulative effect of social, economic and environmental conditions. Ongoing evidence from the Marmot Review demonstrates widening mortality gaps linked to deprivation and insecure work, as austerity and labour market restructuring continue to amplify chronic disease burdens (Marmot and Allen, 2025).

These insights point towards the need for frameworks that move beyond behavioural and molecular logics towards approaches that recognise health as relational, cumulative and ecologically embedded (Hinchliffe et al., 2025). The following section considers how emerging metabolic perspectives offer such a reframing, shifting analytical attention from individual optimisation towards the systemic conditions through which health and inequality are jointly produced.

## Metabolic justice: A new paradigm?

Despite decades of discourse centred on lifestyle modification and, more recently, precision and personalised medicine, health inequalities persist, determined not by choice or genetic predisposition alone, but by enduring conditions of poverty, racialised disadvantage, labour precarity and environmental degradation (Marmot and Allen, 2025). These intersecting conditions disrupt the epistemological dominance of behavioural and molecular logics, demanding a renewed focus on the social production of health.

Metabolic justice is not simply a metaphor, but a framework that foregrounds the material processes through which social, economic and ecological inequalities become biologically embodied. Unlike behavioural or genomic paradigms, which locate responsibility within the individual and their body, metabolic justice situates health within networks of dependency and exposure. As Hinchliffe et al. (2025) argue, metabolism can no longer be understood solely in somatic terms; it is shaped by flows and exposures, including nutritional environments, toxic ecologies, labour conditions and stressors, which accumulate across time and space. Metabolic processes are social and ecological as much as biological, posing new challenges, and opportunities, for justice. What distinguishes metabolic justice from adjacent frameworks such as the social determinants of health, environmental justice or health equity is its insistence on metabolism as both an analytic and political lens. While social determinants approaches identify the structural conditions that shape health, and environmental justice highlights unequal exposures to harm, metabolic justice foregrounds the cumulative, embodied processes through which these inequalities are materially incorporated across time. It thus bridges biological and social analysis by focusing on how flows of nutrients, toxins, stressors and resources are differentially distributed and metabolised within and across populations. In doing so, it shifts attention from discrete risk factors or exposures to the relational and temporal dynamics through which inequality becomes biologically embedded, reframing health not simply as an outcome of structural forces but as their ongoing materialisation within bodies and environments. In this way, metabolic justice shifts analytical focus from the distribution of risk to the processes through which inequality becomes materially inscribed upon, and sedimented within, bodies across lifecourses and generation.

Metabolic injustice arises where social and ecological inequities converge to produce embodied harm, a lived reality that stands in stark contrast to narratives framing health outcomes as the result of individual choice or genetic predisposition. This convergence is not abstract; it manifests in lived realities across diverse contexts. This is evident across multiple contexts. First Nation communities such as the Marind in West Papua have faced the loss of customary forest foods like sago due to corporate land grabs, disrupting traditional diets and ancestral food–environment relations (Firdaus, 2020). Here, colonial and extractive regimes generate metabolic harm across generations. In Flint, Michigan, USA, lead contamination of drinking water poisoned over 100,000 predominantly black residents, including 9000 children, causing irreversible impacts on metabolism, development and long-term wellbeing (Riley, 2024). Similarly, culturally and racially marginalised urban communities across North America experience disproportionate exposure to industrial pollutants and highways, correlating with elevated rates of metabolic syndrome and cardiovascular disease. These cases underscore that metabolic harm is not incidental but structurally produced and embedded within histories of dispossession, environmental racism and systemic neglect (Wilson-Gilmore, 2023).

Metabolic injustice operates through multiple pathways, including the workplace and the environment, where social inequities become biologically inscribed. The Whitehall II cohort study followed 6123 British civil servants over 18 years, finding a 25% lower risk of incident metabolic syndrome among men reporting high levels of organisational justice (Gimeno et al., 2010). Follow-up research (Varga et al., 2022) using clinical biomarkers confirmed that higher organisational justice predicted more favourable long-term metabolic trajectories, lower BMI, triglycerides and insulin. De Vogli et al. (2007) further demonstrated that perceptions of unfairness at work were independently associated with 1.7 times higher odds of developing metabolic syndrome. These dynamics intersect with environmental injustice. For example, in London, UK, ethnic minority and low-income communities face up to 30% higher exposure to air pollution, from major roads or industrial sites, a form of environmental racism that correlates with elevated diabetes and cardiovascular rates (Gadenne et al., 2024). Emerging scholarship in critical metabolism argues that such burdens, ranging from psychosocial stress to environmental toxicity, constitute metabolic injustice, demanding systemic interventions that address food insecurity, housing precarity and ecological degradation (Hinchliffe et al., 2025).

Viewed through this lens, metabolic justice calls for new forms of governance beyond the scope of behavioural interventions or individualised models of care. Rather, it demands regulatory and redistributive approaches capable of addressing the conditions that generate cumulative exposure, including labour exploitation, environmental degradation, broken food systems and housing precarity. Here, institutional arrangements must prioritise accountability for structural harms, rather than responsibilising individuals for managing their outcomes.

However, metabolic justice also presents analytical and political challenges. The multiplicity of exposures involved, and the cumulative, long-term nature of metabolic processes make causal attribution and intervention more complex. Moreover, by foregrounding biological incorporation there is a risk that social inequalities may be re-biologised if not carefully situated within political economy. As a consequence, metabolic justice should not be understood as a comprehensive solution, but as a productive reframing that foregrounds the embodied consequences of structural inequality, while raising difficult questions about governance, responsibility and scale.

In these contexts, justice demands more than individual risk management or access to personalised technologies. It requires transformative policies capable of addressing food insecurity, housing precarity and labour exploitation, ecological repair to mitigate climate-driven health risks and institutional arrangements that hold systems, not individuals, accountable for cumulative harm. These conditions converge within social-ecological metabolisms, reflecting both regional inequities and planetary pressures. As Hinchliffe et al. (2025) emphasise, recognising metabolic injustice means acknowledging how ecosystems and social systems jointly shape metabolic health and how justice must be oriented towards remedying these intertwined harms. To do so requires reconceiving wellbeing as a collective good, challenging market logics that commodify health and promissory narratives that obscure structural violence. By centring redistribution, ecological repair and systemic accountability, metabolic justice has the potential to revive the moral ambition of *Health for All* and its descendants, not as a rhetorical aspiration but as a materially grounded and politically transformative project capable of addressing the cumulative harms of metabolic inequality.

At the same time, metabolic justice is not without its limitations and tensions. As emerging scholarship acknowledges, tracing the pathways through which social and ecological inequalities become metabolically embodied is methodologically complex, often involving diffuse causal chains, long temporal horizons and multiple interacting exposures (Hinchliffe et al., 2025). Questions of political agency and accountability are similarly complicated: if metabolic harm is produced cumulatively across systems and generations, identifying appropriate sites of intervention and responsibility becomes increasingly difficult. There is also a risk that foregrounding metabolism could unintentionally re-biologise social problems if not carefully situated within a critical political economy. Rather than a complete solution, metabolic justice is therefore best understood as a productive reframing, one that opens new analytical and political possibilities while remaining attentive to the practical and epistemological constraints it entails.

## Discussion

This paper has traced the trajectory of health governance from collective responsibility anchored in welfare states and structural reform to an increasingly individualised model shaped by neoliberal logics. Initially expressed through lifestyle and risk discourses, these imperatives have intensified through new discovery science and innovations in molecular and genomic medicine, further producing responsibilised subjects tasked with managing biological futures via data-driven interventions and market-mediated technologies (Kent, 2023). Framed as empowering, these paradigms obscure structural determinants and recast inequality as individual failure. The COVID-19 syndemic exposed the fragility of individualised health paradigms, challenging the reduction of outcomes to behavioural choice or genomic predisposition. Thirty years on from the publication of *Health’s* first issue, this rupture demands a critical reorientation of health scholarship and policy.

Armstrong’s (2025) bibliometric analysis of Crawford’s concept of healthism underscores its historical depth and contemporary resilience. Despite technological shifts, from lifestyle medicine to genomic profiling and algorithmic diagnostics, an underlying logic of health as a moralised project of optimisation and responsibilisation persists. This adaptability confirms Robertson’s (2001) observation that dominant health narratives align with prevailing political and economic orders. Here, technological innovation does not displace healthism but recalibrates it, embedding neoliberal rationalities deeper into the molecular domain. Yet this continuity also exposes a critical fault line. By framing health as an individualised project, these paradigms obscure the structural determinants that shape vulnerability, a reality exposed by the syndemic dynamics of COVID-19 and the widening health gaps documented by a steady flow of evidence of the social determinants of health (Ezell, 2023). For critical health scholarship this signals the need to interrogate not only new biomedical technologies but the normative frameworks that sustain them, frameworks that without structural reorientation, will continue to perpetuate inequality under the guise of empowerment. Earlier efforts to move beyond pathological models of health, such as Antonovsky’s (1996) salutogenic framework, similarly emphasised the conditions that support wellbeing. However, their focus on individual coping and resilience sits uneasily alongside the present argument, which foregrounds how structural inequalities are produced and become materially embodied rather than managed. It is within this context that metabolic justice emerges as a counterpoint, reframing health as relational, cumulative and ecologically embedded rather than reducible to behavioural choice or genomic predisposition.

The challenges are clear. Economic inequality has not only persisted but deepened since the neoliberal turn of the 1980s, as Savage (2021) documents through the rebound of class stratification and Piketty (2014) through the U-shaped trajectory of wealth disparities, with a parallel widening of health that has accelerated sharply after the financial crisis (Bambra, 2024). These global trends in socioeconomic inequalities matter. Health inequalities track broader socioeconomic shifts, with mortality rates during COVID-19 twice as high in deprived neighbourhoods and chronic disease burdens remaining concentrated among the most disadvantaged (Dukhovnov and Barbieri, 2022). Even so, despite these dynamics, patient groups, communities and social movements have contested and reworked forms of responsibilisation, articulating alternative visions of collective health (Epstein and Timmermans, 2021).

What next for health justice in an increasingly chaotic world? Focusing upon metabolic justice offers one pathway. By conceptualising health as relational, cumulative and ecologically embedded, it foregrounds redistribution, reparative accountability and systemic reform. Unlike behavioural or genomic paradigms that locate responsibility within the individual body, metabolic justice situates health within networks of exposure and dependency; flows of energy, resources and toxins shaped by political economy and ecological degradation (Hinchliffe et al., 2025). Most importantly, it reframes wellbeing as a collective good, challenging market logics that commodify health and promissory narratives that obscure structural violence. In doing so, it revives the ideals of *Health for All* (and its descendants) some 40 years on, not as a rhetorical aspiration but as a materially grounded, politically transformative ambition, capable of addressing the slow, cumulative harms of metabolic inequality and the intersecting crises of climate, labour and social reproduction.

As *Health* reaches its 30th anniversary, it is imperative that future research and scholarship continues to operationalise this framework, interrogating how governance, markets and technologies can be reconfigured to support collective wellbeing rather than individual optimisation. This demands moving beyond the epistemological dominance of risk and responsibilisation that have persisted in lifestyle and molecular discourses, towards approaches that recognise health as a social and ecological project, embedded in social justice rather than individual choice.

## Notes

1. Market reports from the Global Wellness Institute and Fitt/Insider (2025) highlight how medspas, neurotoxins, GLP-1–influenced body shaping, and ‘Instagram face’ aesthetics are framed as extensions of health under this logic of optimisation.
